# Thermospermine Synthase (*ACL5*) and Diamine Oxidase (*DAO*) Expression Is Needed for Zygotic Embryogenesis and Vascular Development in Scots Pine

**DOI:** 10.3389/fpls.2019.01600

**Published:** 2019-12-20

**Authors:** Jaana Vuosku, Riina Muilu-Mäkelä, Komlan Avia, Marko Suokas, Johanna Kestilä, Esa Läärä, Hely Häggman, Outi Savolainen, Tytti Sarjala

**Affiliations:** ^1^Ecology and Genetics Research Unit, University of Oulu, Oulu, Finland; ^2^Production Systems, Natural Resources Institute Finland, Espoo, Finland; ^3^Research Unit of Mathematical Sciences, University of Oulu, Oulu, Finland

**Keywords:** arginine decarboxylase, developmental regulation, diamine oxidase, enzyme pathway evolution, polyamine, Scots pine, thermospermine synthase, zygotic embryogenesis

## Abstract

Unlike in flowering plants, the detailed roles of the enzymes in the polyamine (PA) pathway in conifers are poorly known. We explored the sequence conservation of the PA biosynthetic genes and diamine oxidase (*DAO*) in conifers and flowering plants to reveal the potential functional diversification of the enzymes between the plant lineages. The expression of the genes showing different selective constraints was studied in Scots pine zygotic embryogenesis and early seedling development. We found that the arginine decarboxylase pathway is strongly preferred in putrescine production in the Scots pine as well as generally in conifers and that the reduced use of ornithine decarboxylase (ODC) has led to relaxed purifying selection in *ODC* genes. Thermospermine synthase (*ACL5*) genes evolve under strong purifying selection in conifers and the *DAO* gene is also highly conserved in pines. In developing Scots pine seeds, the expression of both *ACL5* and *DAO* increased as embryogenesis proceeded. Strong *ACL5* expression was present in the procambial cells of the embryo and in the megagametophyte cells destined to die *via* morphologically necrotic cell death. Thus, the high sequence conservation of *ACL5* genes in conifers may indicate the necessity of *ACL5* for both embryogenesis and vascular development. Moreover, the result suggests the involvement of *ACL5* in morphologically necrotic cell death and supports the view of the genetic regulation of necrosis in Scots pine embryogenesis and in plant development. *DAO* transcripts were located close to the cell walls and between the walls of adjacent cells in Scots pine zygotic embryos and in the roots of young seedlings. We propose that DAO, in addition to the role in Put oxidation for providing H_2_O_2_ during the cell-wall structural processes, may also participate in cell-to-cell communication at the mRNA level. To conclude, our findings indicate that the PA pathway of Scots pines possesses several special functional characteristics which differ from those of flowering plants.

## Introduction

Polyamines (PAs), ancient small polycations, are found in all living organisms (Michael, 2016). The most common PAs in eukaryotic cells are putrescine (Put), spermidine (Spd), and spermine (Spm) (Tiburcio et al., 1997; Pegg, 2009). Other PAs include cadaverine (Cad) (Bagni and Tassoni, 2001) and thermospermine (T-Spm), a structural isomer of Spm, which is widely found in the plant kingdom (Knott et al., 2007; Takano et al., 2012). PAs show high affinity for polyanionic macromolecules, such as DNA, RNA, proteins, and phospholipids, and function in various fundamental cellular processes, such as DNA and protein syntheses, gene expression, cell division and elongation, differentiation, free radical scavenging, and programmed cell death (Ha et al., 1998; Childs et al., 2003; Seiler and Raul, 2005; Igarashi and Kashiwagi, 2010; Schuster and Bernhardt, 2011). In plants PAs are involved in numerous physiological events as well as different abiotic and biotic stress responses (Galston and Sawhney, 1990; Kumar et al., 1997; Bouchereau et al., 1999; Alcázar et al., 2006; Kusano et al., 2008) during which PA homeostasis is achieved by modulating PA biosynthesis, catabolism, conjugation, and transport (Tiburcio et al., 2014).

In plants PAs are biosynthesized and catabolized *via* branched enzymatic pathways (**Supplementary Figure S1**). Ornithine decarboxylase (ODC) produces Put directly from ornithine. In addition, plants generally possess an additional route for Put formation from arginine consisting of the enzymes arginine decarboxylase (ADC), agmatine iminohydrolase (AIH), and N-carbamoylputrescine amidohydrolase (CPA) (Tiburcio et al., 1997). Diamine Put is the immediate precursor of the tri- and tetra-amines Spd, Spm, and T-Spm which are synthesized by combined actions of S-adenosylmethionine decarboxylase (SAMDC) and the aminopropyltransferases spermidine synthase (SPDS), spermine synthase (SPMS), and thermospermine synthase (TSPMS) (Shao et al., 2012). Both SPDS and SPMS activities can also occur in the same bifunctional enzyme (Vuosku et al., 2018). Put is catabolized by the action of diamine oxidases (DAOs) belonging to the group of copper-containing diamine oxidases (CuAOs) and the higher PAs by the flavoprotein-containing PA oxidases (PAOs) (Bagni and Tassoni, 2001; Bais and Ravishankar, 2002; Marina et al., 2013) (**Supplementary Figure S1**).

Because Put and Spd are the only PAs produced in all PA-synthesizing eukaryotes, the extant core of the eukaryotic PA biosynthetic pathway in the last eukaryotic common ancestor might have consisted of the ODC and SPDS enzymes (Michael, 2016). In plants the complex evolutionary history of the PA biosynthesis pathway includes the transfers of the ADC pathway from the cyanobacterial ancestor of the chloroplast (Illingworth et al., 2003) and TSPMS encoding *ACL5* genes from archaea or bacteria (Minguet et al., 2008). The duplications of *SPDS* genes have led to the evolution of separate SPDS and SPMS enzymes in flowering plants (Minguet et al., 2008), whereas a bifunctional progenitor enzyme possessing both SPDS and SPMS activity was preserved in an evolutionary old conifer (Vuosku et al., 2018). All plant species, including Scots pine (*Pinus sylvestris* L.) and the moss *Physcomitrella patens* (Hedw.) Bruch & Schimp possess sequences identified as *SPDS* or *ACL5*, but no genomic sequence like the one described for *SPMS* has been reported from gymnosperms or *Physcomitrella* so far (Minguet et al., 2008; Rodríguez-Kessler et al., 2010; Vuosku et al., 2018). Although the synthesis of T-Spm likely resembles the formation of Spm (Knott et al., 2007), the TSPMS and SPMS enzymes have different evolutionary origin—TSPMS being more ancient than SPMS in plants.

The importance of PAs in plant embryogenesis has been documented in both angiosperms and gymnosperms (Baron and Stasolla, 2008). PA biosynthetic knock-out mutants have indicated that ADC (Urano et al., 2005), SPDS (Imai et al., 2004) and SAMDC (Ge et al., 2006) are essential for embryo development in Arabidopsis (*Arabidopsis thaliana* L.). Both *ADC* and *SPDS* expressed in the mitotic cells of Scots pine zygotic embryos, which supports the essential roles of Put and Spd in basic cell functions (Vuosku et al., 2006; Vuosku et al.,2018). In contrast, the Arabidopsis *acl5-1 spms-1* double mutant, which contains neither Spm nor T-Spm, is viable and shows no phenotypic change except for the reduced stem growth due to *acl5-1* (Imai et al., 2004). In conifers PA contents and ratios followed developmental stage dependent profiles during zygotic embryogenesis (Minocha et al., 1999; Astarita et al., 2003; Vuosku et al., 2006; de Oliveira et al., 2017). Likewise, the triggering of somatic embryogenesis pathway modulated the PA metabolism at gene expression, enzyme activity, and metabolite levels (Minocha et al., 1999; Gemperlová et al., 2009; Jo et al., 2014; Salo et al., 2016). However, the reactions of embryogenic cell masses to exogenous PAs and PA biosynthesis inhibitors have proved to be complex and dependent on both the conifer species and the developmental stage of somatic embryos (Santanen and Simola, 1992; Minocha et al., 1993; Sarjala et al., 1997; Kong et al., 1998; Niemi et al., 2002; Steiner et al., 2007).

The extreme evolutionary conservation of PAs indicates their necessity in organism survival. On the other hand, different evolutionary processes as well as the high flexibility of PA metabolism in response to internal and environmental demands, especially in plants, implicate that PAs may have acquired a wide variety of different functions during evolution. *Pinus* species of the gymnosperms present an evolutionarily old group of vascular plants that last shared a common ancestor with angiosperms about 300 million years ago (Zhang et al., 2004). We hypothesized that the enzymes in the PA metabolic pathway have acquired different functions during their evolution after the divergence of the seed plant lineages. This hypothesis was supported by our previous findings showing that the Scots pine has a bifunctional SPDS also possessing SPMS activity, which is contrary to angiosperms, which rely on separate enzymes in Spd and Spm biosynthesis (Vuosku et al., 2018). In the present study we are looking for other specific functional characteristics possibly existing in the PA metabolism of Scots pine. Therefore, we compared the sequence conservation of the PA biosynthesis genes and *DAO* between angiosperms and gymnosperms for screening out dissimilarities that potentially indicate functional diversification of the enzymes (Fay and Wu, 2003). After that, the expression of the genes showing different selective constraints between the plant lineages was examined in Scots pine seeds and seedling tissues. Our findings reveal the preference of the ADC pathway in Put biosynthesis in conifers and emphasize the importance of *ACL5* and *DAO* in development by suggesting novel roles in cellular functions for them. Furthermore, the results underline the strict developmental regulation of the PA metabolism in Scots pine.

## Materials and Methods

### Plant Material

One-year-old immature seed cones were collected from two open-pollinated elite Scots pine (*Pinus sylvestris* L.) clones, K881 and K884, growing in the Scots pine clone collection in Punkaharju, Finland (61°48′ N; 29°17′ E). For both clones one representative graft was selected and used repeatedly for cone collection in different years of the PA research (e.g. Vuosku et al., 2006; Vuosku et al., 2012; Muilu-Mäkelä et al., 2015a; Salo et al., 2016). For the present study, cones were collected four times during the growing season of 2004, on July 5 (sampling date I), July 12 (sampling date II), July 19 (sampling date III), and July 26 (sampling date IV). The effective temperature sums (i.e. the heat sum unit based on the daily mean temperatures minus the adapted +5°C base temperature) were 436.3, 509.1, 587.4, and 678.9 d.d. on sampling dates I, II, III, and IV, respectively. In Scots pines the time of fertilization and, consequently, embryo development vary between years in the same locality according to the effective temperature sum (Sarvas, 1962; Vuosku et al., 2006). The sequence of embryo development is divided into three phases, which include proembryogeny, early embryogeny, and late embryogeny (Singh, 1978). Previously, Scots pine embryos were found to follow a developmental pattern in which a great majority of embryos were still at the early embryogeny stage when the effective temperature sum was between 600 and 700 d.d. (Vuosku et al., 2006), as was the case on sampling date IV in the present study. Immature seeds were removed from the developing cones and seed coats were removed. Each pooled sample contained about 20 seeds. Mature seed cones were collected from clone K884 in late autumn of the same growing season. Seeds were sterilized overnight in 3% Plant Preservative Mixture TM (Plant Cell Technology, USA) and germinated on moist filter papers in petri dishes (100 seeds/petri dish) in a growth chamber (Weiss Technik, type 266532/1/−SKS30058/+5…+45 3 µPa) at +20 °C, in 100% moisture and under continuous light. After 2 days of germination embryos and megagametophytes were sampled and, after 16 days of germination, the cotyledons, hypocotyls, and roots of the seedlings were excised. Samples (consisting of 20 embryos, 10 megagametophytes or needles, hypocotyls, or roots from tree seedlings) were stored in liquid nitrogen for RNA extraction. For the mRNA *in situ* hybridization assays of *ACL5* and *DAO* transcripts immature seeds (without seed coat) and roots were fixed immediately after sampling as described previously in Vuosku et al. (2015).

Seeds collected from 24 open-pollinated (mostly halfsibs) families from three different populations (Kolari, Northern Finland: latitude 67°11′N, 24°03′E; Punkaharju, Southern Finland: latitude 61°48′N, 29°19′E and Radom, Poland: latitude 50°41′N, 20°05′E) were used for the sequencing of *ADC* and *DAO* genes.

### Estimation of Nonsynonymous to Synonymous Substitution Rate Ratios in PA Genes

The nonsynonymous to synonymous substitution rate ratios (Ka/Ks) were used to infer the strength of purifying selection pressure (Hughes and Nei, 1988) on the genes in the PA biosynthesis pathway and the *DAO* genes. High selective constraints and strong purifying selection should result in low numbers of nonsynonymous substitutions (Ka) between species. As the mutation rate varies across the genome, the rate of neutral synonymous substitutions (Ks) will also vary. Thus, the ratio of Ka/Ks provides information on the level of constraint in protein-coding sequences across related species (Fay and Wu, 2003). For Ka/Ks calculations the PA gene sequences were retrieved from NCBI GenBank (**Supplementary Table S1**). Due to the different appearance of the *ODC*, *SPMS*, and *ACL5* genes in plant species, the availability of sequence data, the reliability of sequence annotations and nucleotide substitution saturation between some sequences, the species used in pairwise Ka/Ks calculations varied slightly for different genes. Two pairs consisting of gymnosperm species and four pairs consisting of angiosperm species were used for all genes except *SPMS*. The Ka/Ks ratios for the *ADC*, *AIH*, *CPA*, *SAMDC*, and *SPDS* genes were calculated between the following pairs: Scots pine (*Pinus sylvestris* L.)–white spruce (*Picea glauca* (Moench) Voss), Scots pine–loblolly pine (*Pinus taeda* L.), rice (*Oryza sativa* L.)–sorghum (*Sorghum bicolor* (L.) Moench), Arabidopsis*–*black cottonwood (*Populus trichocarpa* L.), Arabidopsis*–*vine *(Vitis vinifera* L.), and black cottonwood*–*vine. The pairs were the same for the *DAO* genes except that white spruce was replaced with Sitka spruce (*Picea sitchensis* (Bong.) Carr.). Ka/Ks ratios for the *ACL5* genes were calculated between Scots pine–white spruce, Scots pine–loblolly pine, rice–sorghum, Arabidopsis–clementine (*Citrus clementina* Hort. ex Tan), Arabidopsis–apple (*Malus domestica* Borkh.), and clementine–apple. The set of the *ODC* sequence pairs included Scots pine–sitka spruce (*Picea sitchensis* (Bong.) Carr.), Scots pine–loblolly pine, rice*–*sorghum, tobacco (*Nicotiana tabacum* L.)–black cottonwood, black cottonwood–vine, and apple–vine. The five compared sequence pairs for *SPMS* were rice*–*sorghum, Arabidopsis–vine, Arabidopsis–clementine, Arabidopsis–apple, and clementine–apple. The Scots pine *ACL5* (HM236828), *ADC* (HM236823), *AIH* (HM236824), *CPA* (HM236825), DAO (HM236829), *ODC* (HM236831), *SAMDC* (HM236826), and *SPDS* (KX761190) sequences were used in the analyses. In the case of other conifers, where no unigene sequences were available, EST information was acquired by BLAST searches against the Scots pine PA genes and used to reconstruct a contig containing the complete coding sequence. The sequences were aligned based on codon boundaries i.e. they were translated to amino acid sequences, aligned, and then transformed back to DNA. Synonymous and non-synonymous changes were calculated with MEGA 5.05 using the Pamilo-Bianchi method (Pamilo and Bianchi, 1993).

### Sequencing of *ADC* and *DAO* Genes and Estimation of Gene Copy Numbers

Genomic DNA was extracted from the haploid megagametophyte tissue of the seeds using the Nucleospin Plant II kit (Macherey-Nagel, Germany) with the lysis buffer PL1. DNA quality was checked by agarose gel electrophoresis and DNA quantity was measured using a NanoDrop ND1000 spectrophotometer (NanoDrop Technologies, USA). All the PCR fragments of a gene were amplified from the same DNA template which was extracted from a single megagametophyte. The PCR products were purified with the MinElute PCR purification kit (Qiagen, USA) or the FastAP Thermosensitive Alkaline Phosphatase and Exonuclease I (Fermentas, Lithuania). The Genome Walker Universal kit (Clontech, USA) was used according to the manufacturer’s instructions to increase the length of the sequenced region to the promoter. Sequencing reactions were carried out with an ABI Prism 3730 DNA Analyzer (Applied Biosystems) with a Big Dye Terminator kit v3.1 (Applied Biosystems). The obtained sequences were verified and edited manually using Sequencher 4.7 (Gene Codes Corporation, USA). The sequencing primers are presented in the **Supplementary Table S2**.

For estimating the *ADC*, *ODC*, and *DAO* gene copy numbers in pine genomes the complete cDNA sequence for *ODC* and the obtained full gene nucleotide sequences for *ADC* and *DAO* were used for BLAST searches against the complete genome of loblolly pine *via* the website http://congenie.org/blast. BLAST searches were also performed using the predicted protein sequences of the *ADC*, *ODC*, and *DAO* genes.

### Phylogenetic Analysis

The multiple CuAO protein alignment (**Supplementary Figure S2**) was created using MUSCLE (Edgar, 2004) in Geneious version 11.1.5 (created by Biomatters, available from http://www.geneious.com). The phylogenetic tree was constructed with maximum likelihood using PhyML 3.3.20180621 (Guindon et al., 2010) as a plug-in in Geneious. The analysis was run with the default parameters, using the Jones, Taylor, and Thornton substitution matrix for protein sequences (Jones et al., 1992) and optimization on tree topology, branch length, and substitution rate. Branch support was obtained by 500 bootstrap replicates.

### RNA Extraction and Reverse Transcription

Total RNA was extracted from the Scots pine tissues using the KingFisher™ mL method (Thermo Electron Corporation, Finland) with the MagExtractor^®^ total RNA purification kit (Toyobo, Japan) according to the manufacturer’s instructions. The samples consisting of immature seeds, mature embryos, megagametophytes, needles, stems, or roots were homogenized in liquid nitrogen using a mortar and pestle and 30 mg of the powder was subsequently used for RNA extraction. The RNA samples were treated with RNase-free DNase (Invitrogen, USA) at RT for 15 min for the elimination of contaminating genomic DNA. Thereafter, the RNA samples were purified with the NucleoSpin^®^ RNA Clean-Up kit (Macherey-Nagel, Germany). The RNA yields were measured three times with OD_260_ analysis using NanoDrop ND1000 spectrophotometer (NanoDrop Technologies, USA) and 1 µg of each RNA sample was subsequently used for the cDNA synthesis. cDNA was reverse-transcribed from an anchored oligo-dT primer by SuperScript II reverse transcriptase (Invitrogen, USA) using standard methods in a reaction volume of 20 µl. PCR with actin (*ACT*) primers, which have been designed so that the amplicon contains an intron, was used for revealing possible genomic contamination in the cDNA samples (Jaakola et al., 2004).

### Quantitative PCR Analysis of mRNA Transcripts

The absolute quantification of *ADC* and *ODC* mRNA transcripts in developing zygotic embryos at early (n = 5) and late (n = 4) developmental stages, in mature embryos (n = 5) and megagametophytes (n = 4) as well as in cotyledons (n = 7), hypocotyls (n = 8), and roots (n = 5) was performed with quantitative real-time PCR (qPCR) analysis using synthesized RNA molecules as standards. The DNA templates from which the RNA molecules could be transcribed were amplified by basic PCR procedure. The PCR primers for the ADC standard were 5′-AGAAATTGGGGATGCTGGAT-3′ and 5′-GCCATCACCGACTGGTATTCACC-3′ and for the ODC standard 5′-TTGCGTTGCAGACGTATTTC-3′ and 5′-CAGCGCAAAAGGACGTAGAT-3′. The upstream primers contained T7 promoter sequence (TAATACGACTCACTATAGGG) and the downstream primers contained a poly (T) tail at their 5′ end, which enabled both the synthesis of RNA molecules and the reverse transcription (RT) of synthesized RNA molecules to cDNA with anchored oligo-dT primers. The DNA molecules were used as templates for *in vitro* transcription by T7 RNA polymerase. The standard curves were generated using serial 10-fold dilutions of synthesized RNA molecules to control variability during both the RT and PCR steps of the analysis. The number of RNA molecules added to the RT reaction in the first standard was 10^11^. The weight of standard RNA (in ng) equivalent to 10^11^ was calculated using the molecular weight of oligonucleotide and Avogadro’s constant (6.022 · 10^23^ mol^−1^). The quantification of the target mRNA (reverse transcribed to cDNA) was based on a standard curve constructed in the same quantification assay. For the relative quantification of *ACL5*, *ADC*, *AIH*, *CPA*, *DAO, SAMDC*, and *SPDS* expression during seed development in Scots pine clones K818 and K884 five biological replicates per clone and per sampling date were performed except for sampling dates III and IV when there were only two and four biological replicates for K884, respectively. The geometric mean (Vandesompele et al., 2002) of three independently regulated reference genes actin (*ACT*), ubiquitin (*UBI*), and glyceraldehyde-3-phosphate dehydrogenase (*GAPDH*) from different functional classes was used for the normalization of the gene expression levels according to the Pfaffl method (2001).

The PCR amplification conditions of the gene fragments were optimized for the LightCycler^®^ 2.0 instrument (Roche Diagnostics, Germany), and the subsequent PCR runs showed a single PCR product in melting curve analysis (The Tm Calling Analysis of Lightcycler^®^ 480 Software release 1.5.0 SP3) and agarose gel electrophoresis. The real-time PCR amplifications were performed using the LightCycler^®^ 480 SYBR Green I Master (Roche Diagnostics, Germany), 50 nM gene specific primers (**Supplementary Table S3**), and 2 μl cDNA in the reaction volume of 20 μl. The real-time PCR amplification was initiated by incubation at 95 °C for 10 min followed by 45 cycles: 10 s at 95 °C, 10 s at 58 °C, and 5 s at 72 °C. Two technical replicates of each PCR reaction were performed to control for the variability of PCR amplification. The Abs Quant/2nd Derivative analysis of Lightcycler^®^ 480 software release 1.5.0 SP3 was utilized to generate the crossing point and concentration values for each sample.

### Localization of *ACL5* and *DAO* Transcripts by mRNA *In Situ* Hybridization

The localization of *ACL5* and *DAO* transcripts was performed by mRNA *in situ* hybridization assay as previously described in Vuosku et al. (2015). The 332-bp long *ACL5* probe was amplified with the primers 5′-GCCGAGCTCGAGAGTAGAGA-3′ (upstream) and 5′-TCGATTTCTTCGGCGTCTAT-3′ (downstream). The primers used for the synthesis of a 342 bp probe for *DAO* transcripts were 5′-ATTTCAGGCATGGAGATTCG-3′ (upstream) and 5′-ATTCTTCACCGTTTGCTTGG-3′ (downstream). The DIG-labeled probes were detected by Anti-DIG-AP Fab fragments and NBT/BCIP substrate (Dig Nucleic acid detection Kit, Roche Molecular Biochemicals, Germany). The sections were examined with a light microscope (Nikon Optiphot 2, Japan) and imaged with an Infinity*1* **-***3*C camera (Lumenera Corporation, Canada) using the IMT iSolution Lite image-processing program (IMT i-Solution Inc., Canada). Adobe Photoshop CS5 was used to adjust contrast, brightness, and color uniformly to entire images.

### Statistical Analysis

The effects of the four sampling dates, the two clones and their interactions on the relative gene expression of the *ADC*, *AIH*, *CPA*, *DAO, SAMDC*, *SPDS*, and *ACL5* genes, each in turn, were analyzed as follows. Because the distribution of the relative gene expression values was highly skewed to the right, we first transformed them to the logarithmic scale. We then fitted a linear regression model on the log-transformed relative gene expression for each gene separately. The sampling date and clone were included as categorical factors with fixed effects, such that their reference levels were sampling date I and clone K818, respectively. Thus, our model is equivalent to a two-way analysis of variance model with interaction. The models were fitted using the function lm (Chambers, 1992) of the R environment version 3.6.1 (R Core Team 2019). The results from estimation of the model parameters on the log-scale were back-transformed onto the original scale, and are presented in terms of the relative contrasts of relative gene expression associated with the levels of the two factors vs. the reference level of that factor, and supplemented with the 95% confidence intervals for the respective contrasts.

## Results

### Fast- and Slow-Evolving Genes in Scots Pine PA Biosynthesis Pathway

**Figure 1:**
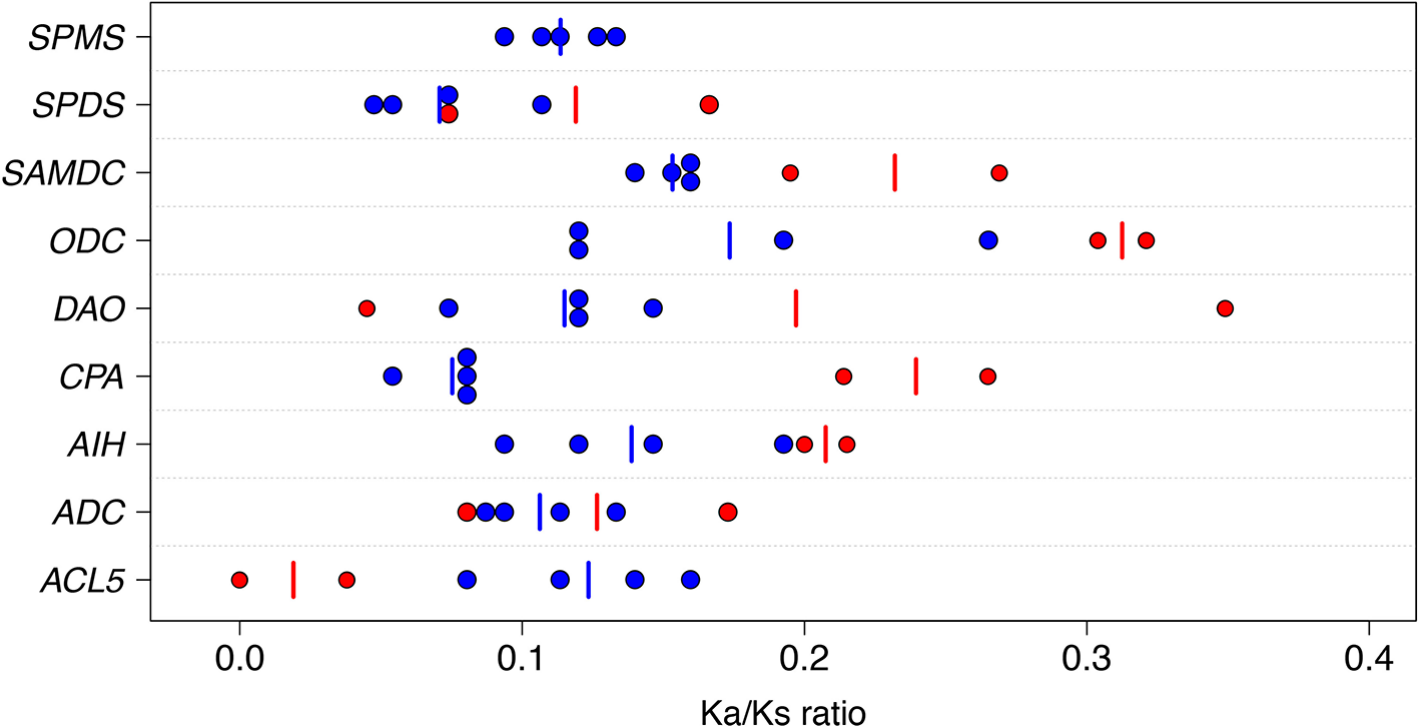
Graphical presentation of the ratios (Ka/Ks) of the nonsynonymous substitution rate (Ka) to the synonymous substitution rate (Ks) in the polyamine genes. Ka/Ks ratios were estimated for the thermospermine synthase (*ACL5*), arginine decarboxylase (*ADC*), agmatine iminohydrolase (*AIH*), N-carbamoylputrescine amidohydrolase (*CPA*), diamine oxidase (*DAO*), ornithine decarboxylase (*ODC*), S-adenosyl methionine decarboxylase (*SAMDC*), spermidine synthase (*SPDS*), and spermine synthase (*SPMS*) genes. Each dot refers to a single pair of angiosperms or gymnosperm species in the comparison. The pairs consisting of angiosperm species are marked with blue symbols and the pairs consisting of gymnosperm species with red symbols. The vertical blue and red line segments indicate the arithmetic mean values of Ka/Ks in angiosperms and gymnosperms, respectively. The angiosperms used for the comparisons were apple, Arabidopsis, black cottonwood, clementine, rice, sorghum, tobacco, and vine. The gymnosperms were Scots pine, loblolly pine, white spruce, and Sitka spruce (see **Supplementary Table S4** for details).

The sequence conservation and the type of selection operating on the protein coding regions of the *ACL5, ADC*, *AIH*, *CPA*, *DAO*, *SAMDC*, *SPDS*, and *SPMS* were studied using the ratio of Ka/Ks, which provides information on the level of evolutionary constraint across related species (Fay and Wu, 2003). Under neutral evolution Ka equals Ks, whereas a low Ka/Ks ratio indicates purifying (negative) selection and a high Ka/Ks ratio directional (positive) selection. Over the PA genes and the pairs of plant species the arithmetic mean values (ranges in parentheses) of Ks, Ka, and Ka/Ks were 0.89 (0.004 to 2.83), 0.10 (0, 0.33), and 0.14 (0, 0.35), respectively (**Supplementary Table S4**). The wide range of Ks estimates reflects the range of very close and very distant phylogenetic comparisons included. There were no sequence pairs with Ka/Ks value greater than one and thus no strong evidence for the contribution of positive selection to the interspecific sequence divergence over the whole gene on any of the PA genes. The highest Ka/Ks values in the PA biosynthesis pathway, 0.32 and 0.30, were found when the Scots pine *ODC* sequence was compared with the Sitka spruce (*Picea sitchensis* (Bong.) Carr.) and loblolly pine (*Pinus taeda* L.) *ODC* sequences, respectively. No nonsynonymous substitutions were found between the Scots pine and loblolly pine *ACL5* sequences leading to the lowest Ka/Ks value. Interestingly, both the highest and lowest Ka/Ks values between the *DAO* sequences, 0.35 and 0.05, were detected in conifers: the Scots pine *DAO* sequence compared to the Sitka spruce and loblolly pine *DAO* sequences respectively (**Figure 1**). Note that the comparisons between the two pine species were based on rather low numbers of substitutions, as average Ks estimates were less than 0.05 (**Supplementary Table S4**).

**Figure 2:**
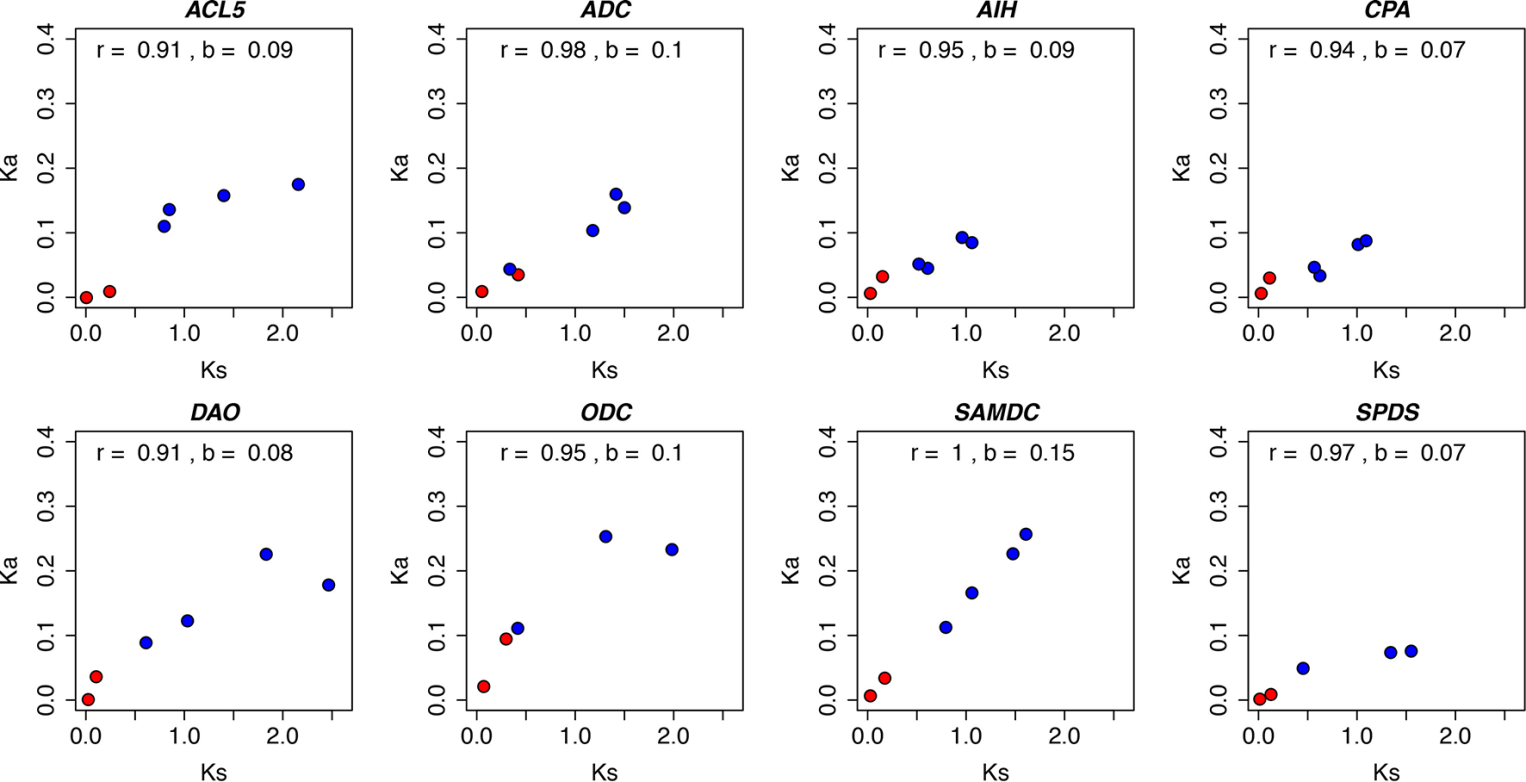
The relationship between nucleotide substitutions at synonymous (Ks) and nonsynonymous (Ka) sites in polyamine biosynthesis genes. Ks and Ka values were estimated for the thermospermine synthase (*ACL5*), arginine decarboxylase (ADC), agmatine iminohydrolase (AIH), N-carbamoylputrescine amidohydrolase (*CPA*), ornithine decarboxylase (*ODC*), S-adenosyl methionine decarboxylase (*SAMDC*), spermidine synthase (*SPDS*), and spermine synthase (*SPMS*) genes. The compared plant pairs consisting of angiosperm species (Arabidopsis, black cottonwood, clementine, rice, sorghum, tobacco, and vine) are marked with blue symbols and the pairs consisting of gymnosperm species (loblolly pine, Scots pine, Sitka spruce, and white spruce) with red symbols (see **Supplementary Table S4** for details). r, correlation coefficient; b, slope of the fitted regression line.

Despite their position in the pathway or evolutionary origin, all the PA biosynthesis genes showed evidence of strong purifying selection across evolutionary time, as their Ka values were much lower than Ks values (**Figure 1** and **Supplementary Table S4**). However, the heterogeneity of the regression coefficients of Ka over Ks (b) suggested different substitution rates at the amino acid level, and thus different strength of purifying selection. The *SAMDC* gene had the highest rate of amino acid change, (b = 0.15), whereas the substitution rate appeared lowest (b = 0.07) for the *CPA* and *SPDS* genes (**Figure 2**). Strong positive correlations were observed between Ka and Ks in all the PA biosynthesis genes with correlation coefficients ranging from 0.91 to 1. The high correlations for individual genes could be a result of the same mutation rates influencing both Ka and Ks. Moreover, due to the very long evolutionary time scales (especially within the angiosperm comparisons), the average strength of purifying selection may have been similar in different lineages (Mouchiroud et al., 1995; Ohta and Ina, 1995). Note that the comparisons between conifers represent very short evolutionary time scale and the positive correlations are mainly driven by the longer-term comparisons of angiosperms.

### Preference of ADC Pathway in Putrescine Biosynthesis in Conifers

The sequencing of Scots pine *ADC* in 24 samples showed that the gene structure is very simple. *PsADC* (HM236823) is an intron-lacking gene composed of a single exon of 2139 bp. BLAST searches against the loblolly pine genome retrieved one full gene sequence for both *ADC* (protein sequence: lcl|PITA_000014798 and nucleotide sequence: lcl|tscaffold9160) and *ODC* (protein sequence: lcl|PITA_000064865 and nucleotide sequence: lcl|tscaffold2337) suggesting that *ADC* and *ODC* are single-copy genes in pine genomes.

**Figure 3:**
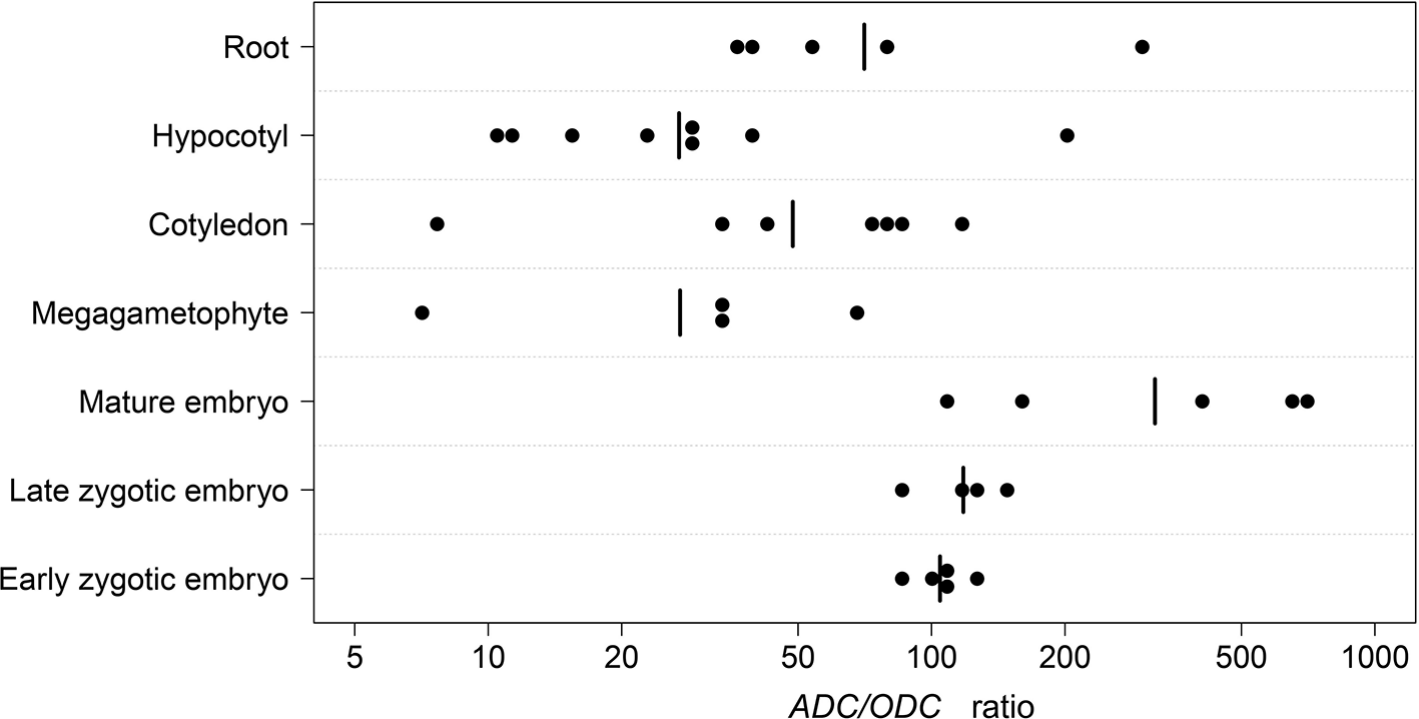
The ratios of the arginine decarboxylase (*ADC*) and ornithine decarboxylase (*ODC*) mRNA transcripts in Scots pine tissues. The number of *ADC* and *ODC* mRNA transcripts was measured in developing zygotic embryos at early (n = 5) and late (n = 4) developmental stages, in mature embryos (n = 5) and megagametophytes (n = 4) as well as in cotyledons (n = 7), hypocotyls (n = 8), and roots (n = 5) of young seedling. The dots represent the *ADC*/*ODC* ratios in the samples (see **Supplementary Figure S3** for further information). n, number of biological replicates.

We hypothesized that the observed differences in the strength of purifying selection acting on the *ADC* and *ODC* sequences in conifers reflect the different use of the ADC and ODC for Put production. To test the hypothesis in the Scots pine, we determined the mRNA copy numbers of the *ADC* and *ODC* genes in developing seeds, mature embryos, and megagametophytes as well as in the cotyledons, hypocotyls, and roots of young seedlings (**Supplementary Figure S3**). The ratios of the *ADC* and *ODC* mRNA transcripts in the samples were used to evaluate the roles of ADC and ODC in Put production in the Scots pine tissues (**Figure 3**). The lowest *ADC*/*ODC*-ratio, seven, was found in megagametophytes as well as in the cotyledons of young seedlings. The highest ratio of 681 was observed in mature embryos. Over the whole data set the geometric mean of the *ADC*/*ODC*-ratio was 66. Thus, Put was almost solely produced *via* the ADC pathway in both developing and mature embryos, but the ADC pathway was also strongly preferred in young seedlings. The Scots pine *ODC* sequence could only be amplified using the second round PCR with hypocotyl cDNA as an original template, which also suggested low expression of the *ODC* gene in the Scots pine tissues. In the BLAST searches against NCBI databases there were more than 100 ESTs from coniferous species, which may represent either homologues or paralogues to the Scots pine *ADC*, whereas there were only 9 ESTs representing *ODC*. The results indicated that the ADC pathway is the principal route for Put biosynthesis in Scots pine but also generally in conifers.

### PA Gene Expression During Scots Pine Zygotic Embryogenesis

**Figure 4:**
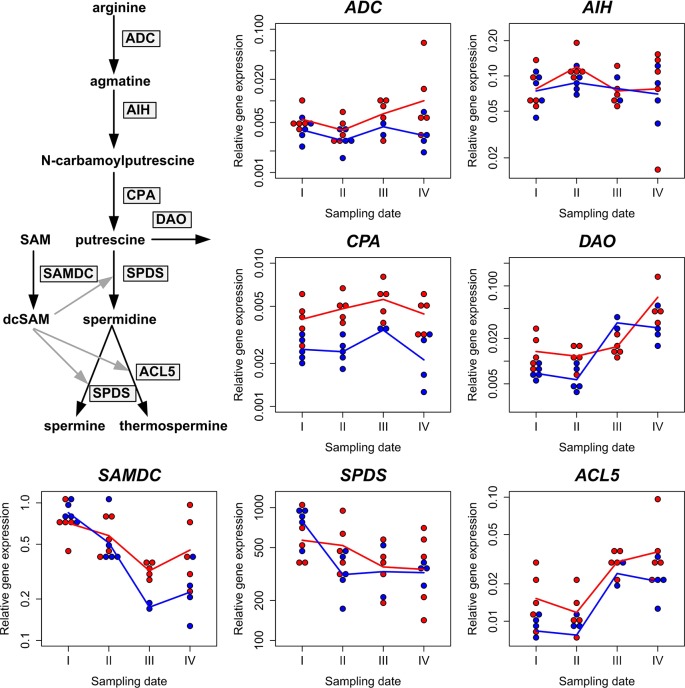
Polyamine gene expression during Scots pine seed development. The relative expression of the arginine decarboxylase (*ADC*), agmatine iminohydrolase (*AIH*), N-carbamoylputrescine amidohydrolase (*CPA*), diamine oxidase (*DAO*), S-adenosyl methionine decarboxylase (*SAMDC*), spermidine synthase (*SPDS*), and thermospermine synthase (*ACL5*) genes is presented relative to the sampling date in clones K818 (red symbols) and K884 (blue symbols). The effective temperature sums were 436.3, 509.1, 587.4, and 678.9 d.d. on sampling dates I, II, III, and IV, respectively. The geometric mean values of the five biological replicates per clone and per sampling date (except for sampling dates III and IV when there are only two and four biological replicates for K884, respectively) are connected with lines. The results of the statistical analyses of these data are presented in **Supplementary Table S5**. Based on them, we found evidence for an increasing trend over time in the expression of *DAO* and *ACL5* in both clones, and some evidence for a decreasing trend in that of *SAMDC*, more pronounced in clone K884. For *CPA*, the gene expression level was consistently higher in K818 than in K884. No other statistically discernible trends or contrasts could be found.

The expression of the *ADC*, *AIH*, *CPA*, *DAO*, *SAMDC, SPDS*, and *ACL5* genes was determined during the Scots pine zygotic embryogenesis (**Figure 4**), which provides a favorable target for studies on PA gene expression in developmental processes due to the simultaneously ongoing cell division, cell specification, and programmed cell death processes (reviewed by Vuosku et al., 2009b). The expression of the genes in the ADC pathway, *ADC*, *AIH*, and *CPA*, remained relatively stable during the embryo development in Scots pine clones K818 and K884. *DAO* expression increased in both clones when the embryo development proceeded. In clone K884 it increased above the value of the baseline (K818, sampling date I) by sampling date III and in clone K818 the value of the baseline was passed by sampling date IV. In the overall decreasing trend of *SAMDC* expression there emerged a difference between the baseline and the sampling date III. *SPDS* expression showed a relatively stable trend in K818 but in K884 a clear drop from the baseline occurred on sampling date II after which the expression stabilized. *ACL5* expression increased with embryo development in both clones and was higher on sampling dates III and IV compared to the baseline (**Figure 4** and **Supplementary Table S5**). Altogether, the results indicated that embryo growth and development was characterized by a consistent increase in both *ACL5* and *DAO* expression, whereas the expression of the genes responsible for the Put biosynthesis remained quite stable during the seed development.

### Association of *ACL5* Expression With Morphologically Necrotic Cell Death

**Figure 5:**
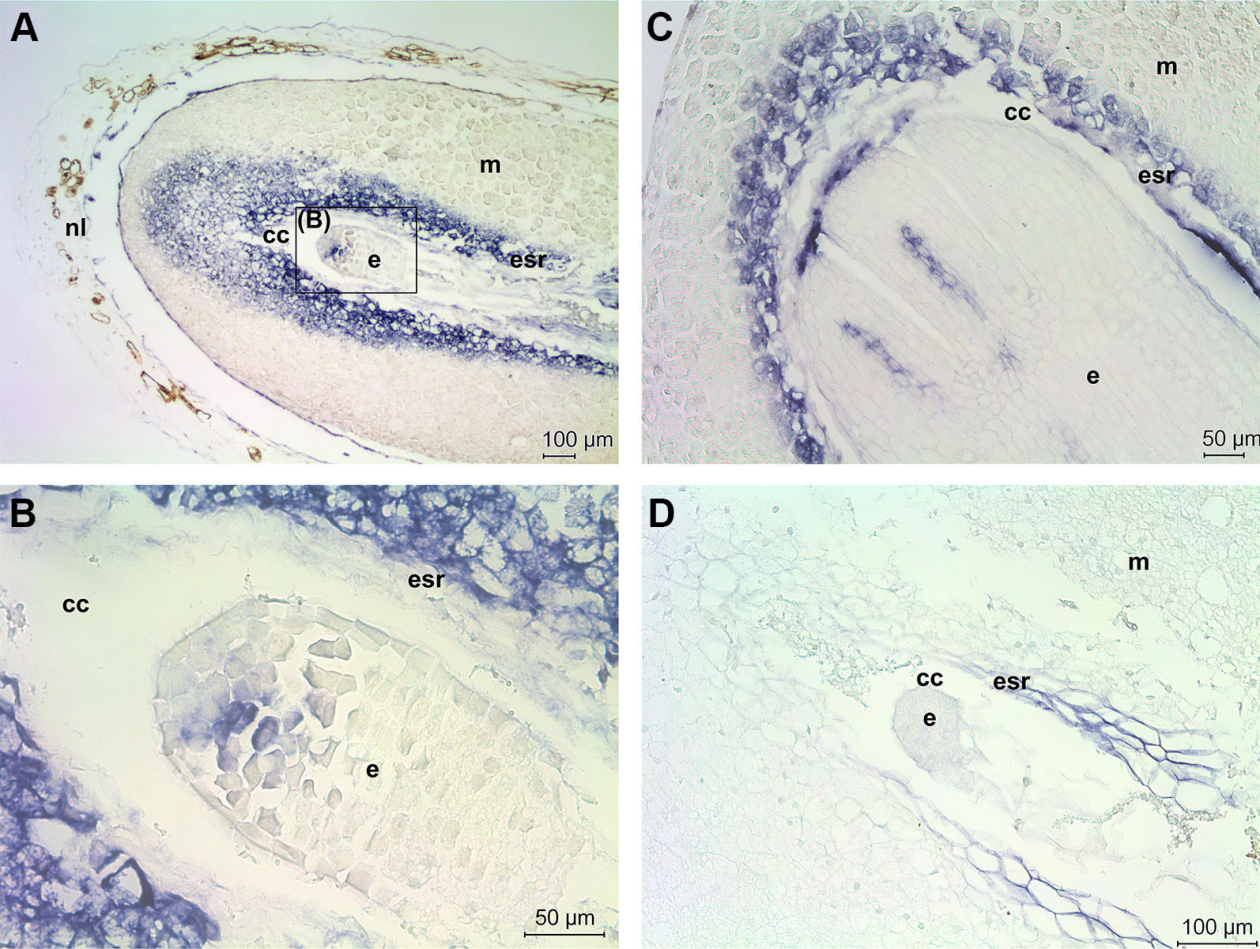
Localization of thermospermine gene (*ACL5*) expression in developing Scots pine seeds. **(A)** *ACL5* expression (blue signal) in the embryo (e) and megagametophyte (m) at the early embryogeny stage. In the megagametophyte, intense *ACL5* expression was localized in the embryo surrounding region (esr) and in the area in the front of the corrosion cavity (cc) **(B)** *ACL5* expression in the specific cells of the early embryo. **(C)** *ACL5* expression in the procambial cells of the embryo and in esr of the megagametophyte at the late embryogeny stage. **(D)** A seed section hybridized with the sense *ACL5* probe as a negative control.

The role of *ACL5* in Scots pine zygotic embryogenesis was studied further by localizing *ACL5* mRNA transcripts. In a developing Scots pine seed the embryo lies within the corrosion cavity of the megagametophyte which houses most of the storage reserves of the seed. The developmental stage of early embryogeny (**Figures 5A**, **B**) initiates with the elongation of the suspensor system and terminates with appearance of the root meristem, whereas late embryogeny (**Figure 5C**) includes the establishment of root and shoot meristems and the maturation of the embryo (Singh, 1978). At the early embryogeny stage, *ACL5* expressed only in few specific cells in the embryo, whereas strong *ACL5* expression was detected in the megagametophyte cells in the embryo surrounding region (ESR) and in the region in the front of the expanding corrosion cavity (**Figures 5A**, **B**). In our previous study we showed that megagametophyte cells in the ESR are destroyed by morphologically necrotic cell death to nourish the developing embryo throughout embryogenesis (Vuosku et al., 2009a). At the late embryogeny stage *ACL5* expressed specifically in the procambial cells of the embryo and in the ESR of the megagametophyte (**Figure 5C**). The specificity of the antisense *ACL5* probe was confirmed by the absence of signals in the seed section hybridized with the sense *ACL5* probe (**Figure 5D**). The non-specific signal observed in the ESR was generated by fragmented nucleic acids as previously described in Vuosku et al. (2010). To conclude, *ACL5* expression was associated with the earliest events of vascular specification in the embryo and with morphologically necrotic cell death in megagametophyte cells.

### Accumulation of *DAO* Transcripts Between the Cell Walls of Adjacent Cells

**Figure 6:**
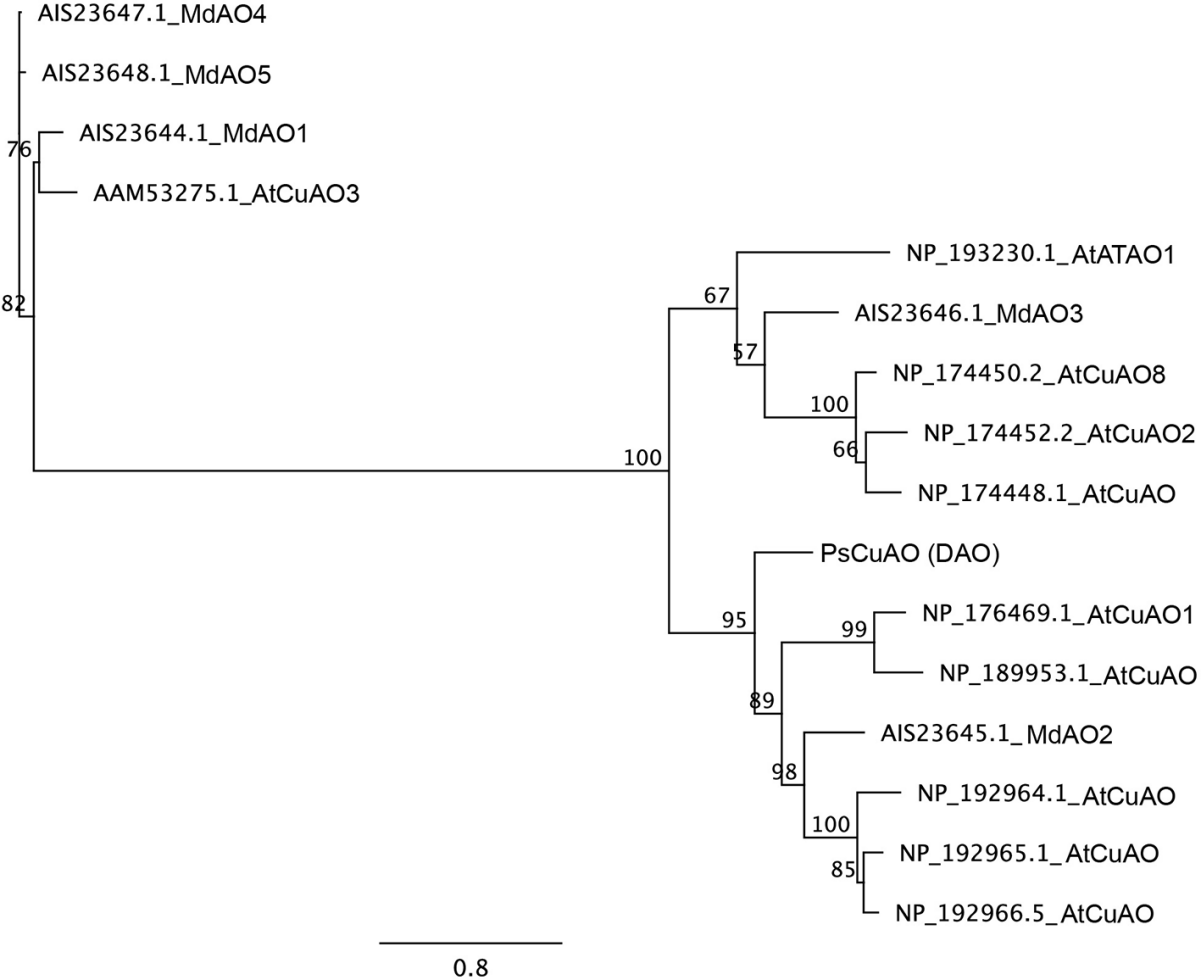
Maximum likelihood phylogeny of CuAO amino acid sequences of Arabidopsis (*Arabidopsis thaliana*), apple (*Malus domestica*), and Scots pine (*Pinus sylvestris*). The tree branch support was obtained by 500 bootstrap replicates. The scale indicates the number of amino acid substitutions per site.

Sequencing of Scots pine putative *PsCuAO* (HM236829), here called *DAO*, in 24 samples showed that the gene is composed of five exons (exon1: 1320 bp, exon2: 114 bp, exon3: 450 bp, exon4: 98 bp, and exon5: 208 bp). The introns are relatively short, with the exception of the first one (intron1: 1240 to 1265 bp, intron2: 106 bp, intron3: 307 bp, and intron4: 394 bp), leading to a total gene size between 4245 and 4262 bp. The coding sequence of the *PsCuAO* gene is 2190 bp long and the predicted protein of 729 amino acids contains the domain structure of the copper-containing amine oxidases. BLAST searches against the loblolly pine genome retrieved only a single full gene copy of *DAO* (protein sequence: lcl|PITA_000016951 and nucleotide sequence: lcl|tscaffold4097). In the phylogenetic analysis of the Scots pine, Arabidopsis and apple CuAO amino acid sequences Scots pine DAO belonged to the same main group with the Arabidopsis CuAO1 and apple MdAO2 proteins with 100% bootstrap support (**Figure 6**).

**Figure 7:**
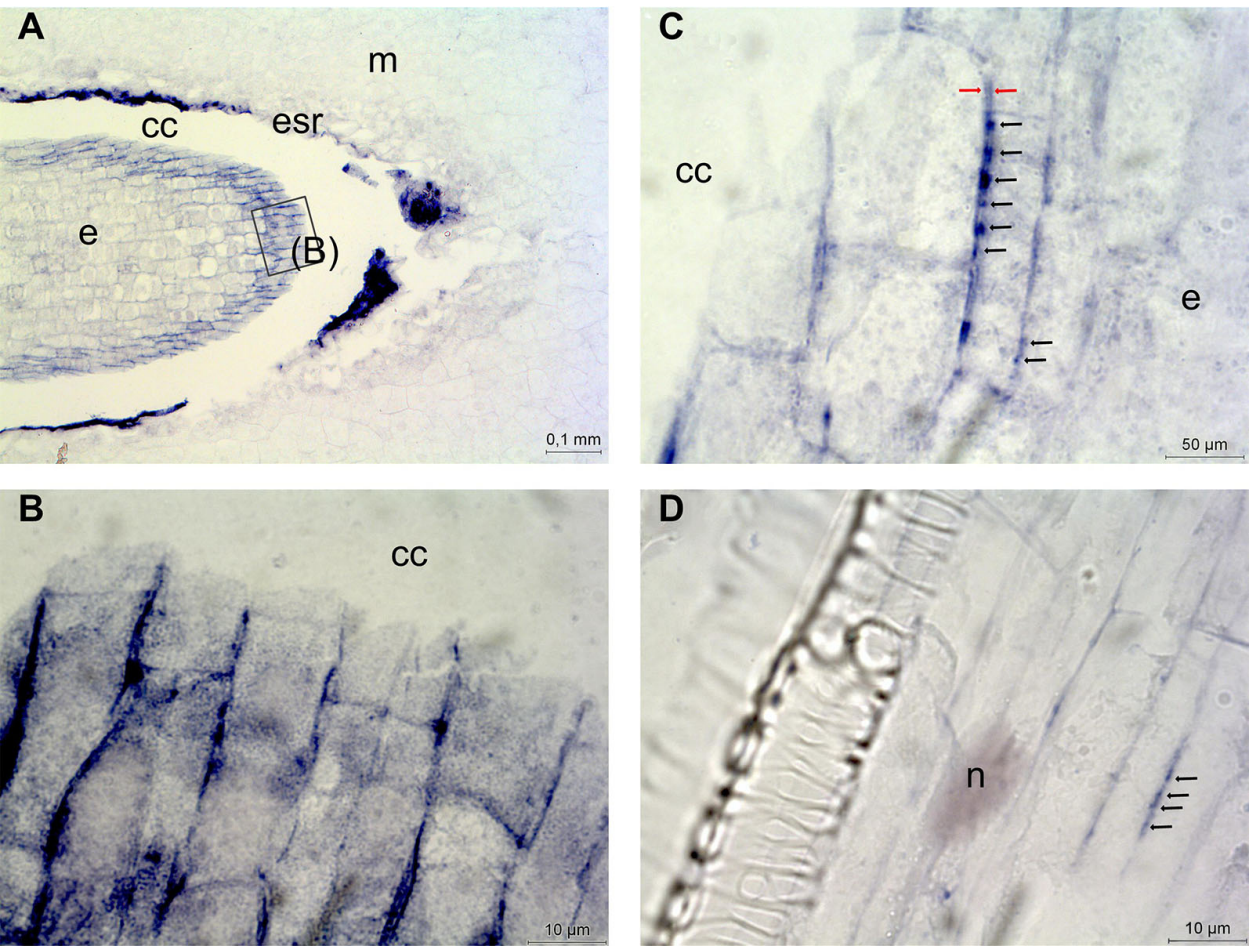
Localization of diamine oxidase (*DAO*) gene expression in Scots pine seeds and roots. **(A)** *DAO* expression in the outermost cells of the early embryo. **(B)** *DAO* transcripts in the vicinity of the cell walls in the early embryo. **(C)** Accumulation of *DAO* transcripts into groups (indicated with black arrows) between the cell walls (indicated with red arrows) of neighboring cells. **(D)** Accumulation of *DAO* transcripts into groups (indicated with black arrows) between two adjacent root cells. cc, corrosion cavity; e, embryo; esr, embryo surrounding region; m, megagametophyte; n, nucleus.

For studying the role of *DAO* in the Scots pine zygotic embryogenesis *DAO* mRNA transcripts were localized in developing Scots pine seeds. *DAO* expression was detected only in the outermost cells of the early embryo (**Figure 7A**) in which *DAO* transcripts were localized in the vicinity of the cell walls (**Figure 7B**). At high magnification *DAO* transcripts were also found to accumulate between the cell walls of neighboring cells where the transcript groups were localized with equal distances from each other forming a string-of-pearls-like appearance (**Figure 7C**). *DAO* expression was minor in zygotic embryos. Therefore, we compared the intensity of *DAO* expression in developing and mature embryos, megagametophytes, cotyledons, hypocotyls, and roots to be able to study the interesting observation further in a Scots pine tissue showing stronger *DAO* expression. The highest *DAO* expression was detected in roots, which were also selected as a target for the localization of *DAO* expression. Similar accumulation of *DAO* transcripts between two adjacent cells was detected in roots (**Figure 7D**). The specificity of the antisense *DAO* probe was confirmed by the absence of signals in the seed and root sections hybridized with the sense *DAO* probe (**Supplementary Figure S4**). In conclusion, in Scots pine tissues *DAO* expression was minor and localized very specifically close to cell walls and between the cell walls of adjacent cells.

## Discussion

In the present study the Ka/Ks ratios (0 to 0.35) over all the PA genes and plant species were in line with the Ka/Ks ratios previously found between *A. thaliana* and *Arabidopsis lyrata* (0.21) (Barrier et al., 2003), between *A. thaliana* and Chinese cabbage (*Brassica rapa* ssp. *pekinensis*) (0.14) (Tiffin and Hahn, 2002), between *Pinus* and *Picea* species (0.10–0.15) (Palmé et al., 2008), between Sitka spruce (*Picea sitchensis* (Bong.) Carr.) and loblolly pine (0.31) (Buschiazzo et al., 2012) in angiosperms (0.09–0.13), in gymnosperms (0.17–0.67) (De La Torre et al., 2017), and among higher plants (Embryophytes) (0.21) (Roth and Liberles, 2006). Generally, PA genes have evolved under strong selective constraint in seed plants (about 80% of Ka/Ks estimates were lower than 0.2), which underlines the fundamental role of the PA metabolism. Furthermore, there was no strong evidence for the contribution of positive selection. However, the criterion of Ka/Ks > 1 is very stringent and several studies with large number of genes have suggested that genes with the lowest Ka/Ks values evolve under strong purifying selection pressure, whereas genes with the highest Ka/Ks values may evolve due to positive selection in addition to relaxed constraints (Charlesworth et al., 2001; Tiffin and Hahn, 2002; Palmé et al., 2008). In conifers *ACL5* genes were found to evolve under stronger purifying selection than in flowering plants, whereas in *ODC* genes selection pressure is relaxed in conifers compared to flowering plants.

In a biosynthetic pathway the existence of alternative routes may lead to weaker purifying selection than in the case of a pathway controlled by a single locus (Ohno, 1970). Our results suggest that this applies in the PA biosynthesis pathway of conifers where *ADC* and *ODC* genes have evolved differently. *ADC* genes have undergone stronger purifying selection likely because of the clear preference of the ADC pathway in Put production, whereas *ODC* genes have evolved more freely due to the reduced use of the ODC enzyme. As in conifers, *ADC* is the primary gene involved in Put biosynthesis in some angiosperms, such as Arabidopsis and apple (Hanfrey et al., 2001; Hao et al., 2005), emphasizing the general importance of the ADC pathway in plants. Thus, we propose that Put biosynthesis from ornithine may not be essential for normal plant growth.

Our results suggest that the PA biosynthesis pathway has less flexibility in Scots pine than generally in flowering plants. According the BLAST searches against loblolly pine genome there is only one copy of both the *ADC* and *ODC* genes in pines, although the presence of other copies cannot be definitely excluded due to the imperfect annotation of conifer genomes. Arabidopsis, for example, possesses two paralogues of *ADC* which has allowed the specialization of the *AtADC1* and *AtADC2* genes (Galloway et al., 1998; Hummel et al., 2004). In addition to the production of Put almost solely *via* the ADC pathway Scots pine possesses a single bifunctional SPDS enzyme for both Spd and Spm synthesis (Vuosku et al., 2018). In flowering plants the duplications of *SPDS* genes has led to presence of more than one *SPDS* genes and, further, to the evolution of separate *SPDS* and *SPMS* genes (Minguet et al., 2008). Thus, the different evolution of the enzyme genes in the PA biosynthesis pathway in the two plant lineages may have resulted in increased adaptability of PA homeostasis in flowering plants compared to conifers.

Both *ACL5* and *DAO* expression increased with embryo development. *ACL5* expression was connected to the earliest events of vascular specification in the developing embryo. Furthermore, intense *ACL5* expression was found in the megagametophyte cells destined to die to provide nourishment and space for the growing embryo. Previously, *ACL5* expression was associated with the later development of the vascular structures during the seed germination and early seedlings growth in Scots pine (Vuosku et al., 2018). T-Spm has been identified as a plant growth regulator that represses xylem differentiation and promotes stem elongation by preventing premature death of developing xylem vessel elements in Arabidopsis (Kakehi et al., 2008; Muñiz et al., 2008; Vera-Sirera et al., 2010). However, *ACL5* expression and the presence of T-Spm were recently found in non-vascular land plants and in the unicellular green alga *Chlamydomonas reinhardtii* (Solé-Gil et al., 2019), which suggested that T-Spm plays also other roles in addition to xylem development in vascular plants. Our present results show a novel function for *ACL5* in the morphologically necrotic cell death and support the view of the genetic regulation of necrosis in Scots pine embryogenesis and further in plant development. Furthermore, the results suggest that higher sequence conservation of the *ACL5* genes in conifers compared to flowering plants results from the necessity of *ACL5* for both embryogenesis and vascular development and, therefore, may reflect differences in the roles of *ACL5* in those crucial developmental events between the plant lineages.

Interestingly, only one *DAO* gene was found from the loblolly pine genome, whereas *DAO* genes form large gene families in flowering plants (Tavladoraki et al., 2016). In Arabidopsis, 10 genes have been annotated as *CuAO*s from which *ATAO1*, *AtCuAO1*, *AtCuAO2*, *AtCuAO3*, and *AtCuAO8* have been also characterized at the protein level (Møller and McPherson, 1998; Wimalasekera et al., 2011; Planas-Portell et al., 2013; Groß et al., 2017). Furthermore, five *CuAO* genes were identified from apple fruit cDNA and the two most abundant (*MdAO1* and *MdAO2*) were biochemically characterized (Zarei et al., 2015). In the phylogenetic tree the Scots pine DAO protein belonged to the same main branch as AtCuAO1 and MdAO2 which have been reported to be extracellular proteins (Planas-Portell et al., 2013; Zarei et al., 2015). In Scots pine zygotic embryos and roots *DAO* expression was localized in the vicinity of cell walls and between the cell walls of adjacent cells, where they formed string-of-pearls-like appearances suggesting that *DAO* transcripts accumulated in the plasmodesmata. We therefore suggest that DAO, in addition to the roles in the regulation of cellular Put content and production of hydrogen peroxide (H_2_O_2_) for cell wall loosening/stiffening events (Tavladoraki et al., 2016), may also participate in cell-to-cell communication at the mRNA level. The role of H_2_O_2_ in signaling has received much attention but it has been also studied for its toxic effects (Smirnoff and Arnaud, 2018). Also, mRNAs may not be constrained to their cell of origin but potentially move to and act in other cells and, thus, serve as messengers between cells (Ham and Lucas, 2017; Morris, 2018). In the light of our results, we suggest that *DAO* transcripts may be transported between cells instead of H_2_O_2_ to avoid harmful effects especially in sensitive developing tissues.

In conifers the detailed roles of the enzymes in the PA pathway have been poorly known, which has hindered the development of PA-based applications in forest biotechnology. Our results suggest that the adaptability of PA homeostasis may be restricted in Scots pine and, thus, the manipulation of PA levels may not provide as practical tools for the enhancement of stress tolerance as in many flowering plants (Hussain et al., 2011; Marco et al., 2016). In both Scots pine seedlings and proembryogenic cells the expression of the PA biosynthetic and catabolic genes was down regulated rather than up regulated during drought/osmotic stress, whereas PA contents remained quite stable (Muilu-Mäkelä et al., 2015a; Muilu-Mäkelä et al., 2015b). Therefore, we suggest that PAs may have more potential for biotechnological applications in development related processes such as somatic embryogenesis and the control of wood formation in Scots pine. During the induction of Scots pine somatic embryogenesis, the triggering of the embryo-producing pathway was connected with consistent changes in PA gene expression (Salo et al., 2016). Thus, the specific manipulation of PA gene expression might provide a way to enhance somatic embryo production in recalcitrant Scots pine lines. In the present study *ACL5* expression was associated with the earliest events of vascular specification during the Scots pine zygotic embryogenesis, and *ACL5* expression has been localized in procambial cells also during seed germination and early seedlings growth (Vuosku et al., 2018). The role of auxin as main regulator of vascular differentiation is well documented (Miyashima et al., 2013) and findings on xylem differentiation have proposed a model of complex functional interaction between auxin, T-Spm, and HD-ZIP III genes (Milhinhos et al., 2013; Baima et al., 2014). Furthermore, Yoshimoto et al. (2012) found that the auxin signaling that promotes xylem differentiation is normally limited by SAC51-mediated T-Spm signaling but can be continually stimulated by exogenous auxin analogs in the absence of T-Spm. Thus, the opposite action between T-Spm and auxin seems to be essential for xylem differentiation and the combined use of these growth regulators might provide opportunities to control wood formation in Scots pine.

## Data Availability Statement

All datasets generated/analyzed for this study are included in the manuscript and the supplementary files.

## Author Contributions

TS, HH, and JV conceived the project. JV mainly designed the study, participated in the molecular evolutionary and gene expression analyses, and acted as a principal author of the manuscript. RM-M performed the statistical analyses of the gene expression results and participated in the writing of the manuscript. KA performed the full gene sequencing, the BLAST search analyses, and participated in the writing of the manuscript. MS performed the computational analysis of the molecular evolution data. JK carried out the *in situ* mRNA hybridization analyses. EL created the statistical graphs and performed the statistical analysis of the molecular evolution data. HH participated in the coordination of the study and in the writing of the manuscript. OS provided expertise in the molecular evolutionary analyses and interpretation of the results. TS participated in the coordination of the study and in the writing of the manuscript. All the authors read and approved the final manuscript.

## Funding

The Research was funded by the Academy of Finland (Project 121994 to TS) and by grants from the Finnish Cultural Foundation and the Niemi Foundation (to JV). OS and KA acknowledge EU project Noveltree (FP7211868) and Biocenter Oulu.

## Conflict of Interest

The authors declare that the research was conducted in the absence of any commercial or financial relationships that could be construed as a potential conflict of interest.
