# Supplementary material for: Thermospermine Synthase (ACL5) and Diamine Oxidase (DAO) Expression Is Needed for Zygotic Embryogenesis and Vascular Development in Scots Pine
Source: Front Plant Sci. 2019 Dec 20;10:1600. doi: 10.3389/fpls.2019.01600 (PMC6934065; doi:10.3389/fpls.2019.01600)
Supplement: Supplementary file 1 [file DataSheet_1.pdf]

Supplementary Material

1. Supplementary Figures and Tables

1.1 Supplementary Figures

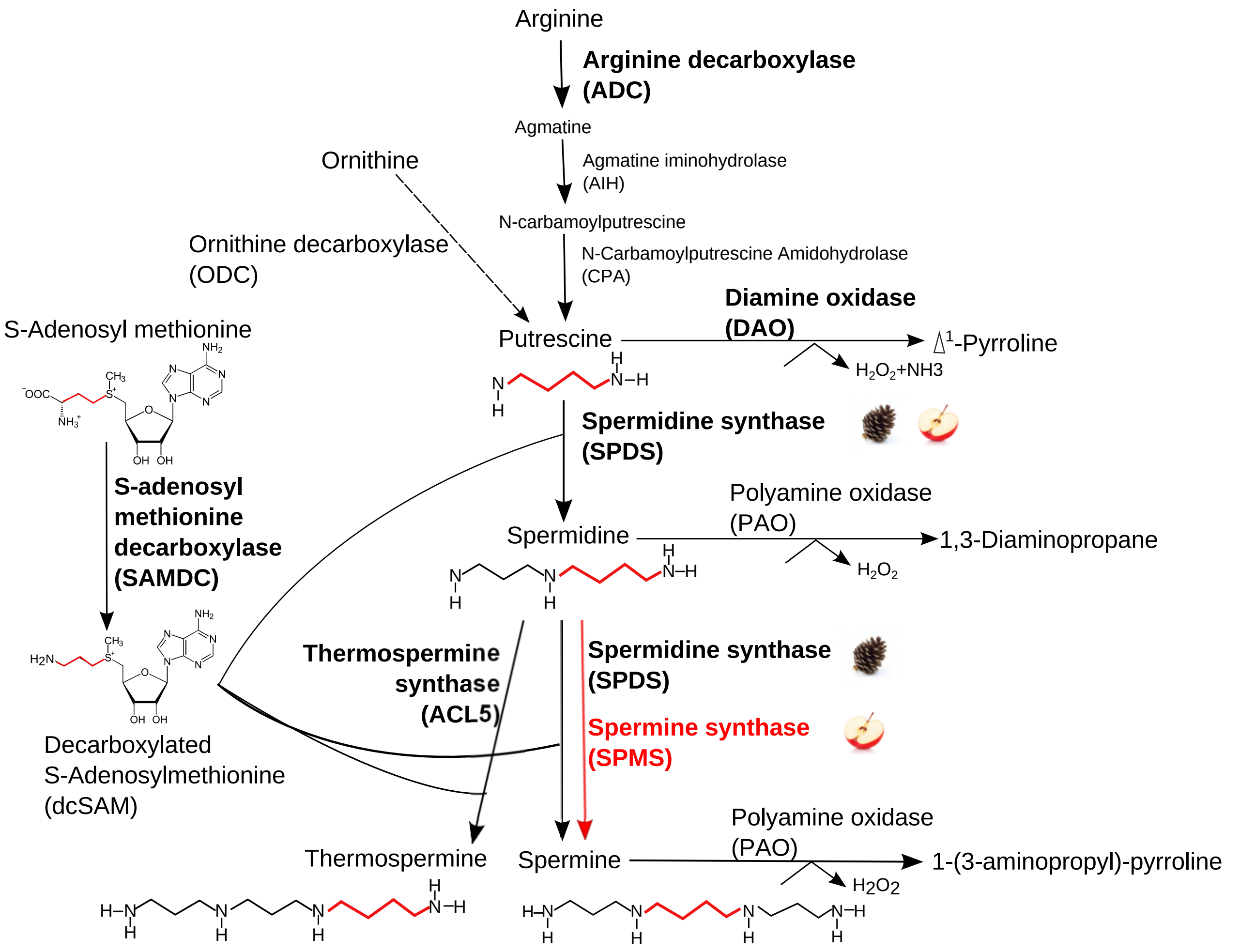

Supplementary Figure S1. Polyamine biosynthesis and catabolism pathways in plants. Diamine putrescine is produced via ADC or ODC from arginine and ornithine, respectively. ADC pathway possesses two intermediate steps (AIH and CPA). Scots pine has a bifunctional SPDS enzyme that adds aminopropyl groups to putrescine and spermidine forming both, spermidine and spermine (highlighted by cones). In angiosperms SPDS produces only spermidine, whereas spermine is produced by SPMS (highlighted by apples and a red arrow). Thermospermine, a structural isomer of spermine, is produced by ACL5. Aminopropyl groups are detached from S-adenosylmethionine by SAMDC. Putrescine is oxidized by DAO whereas higher polyamines spermidine, spermine and thermospermine are catabolized by PAO.

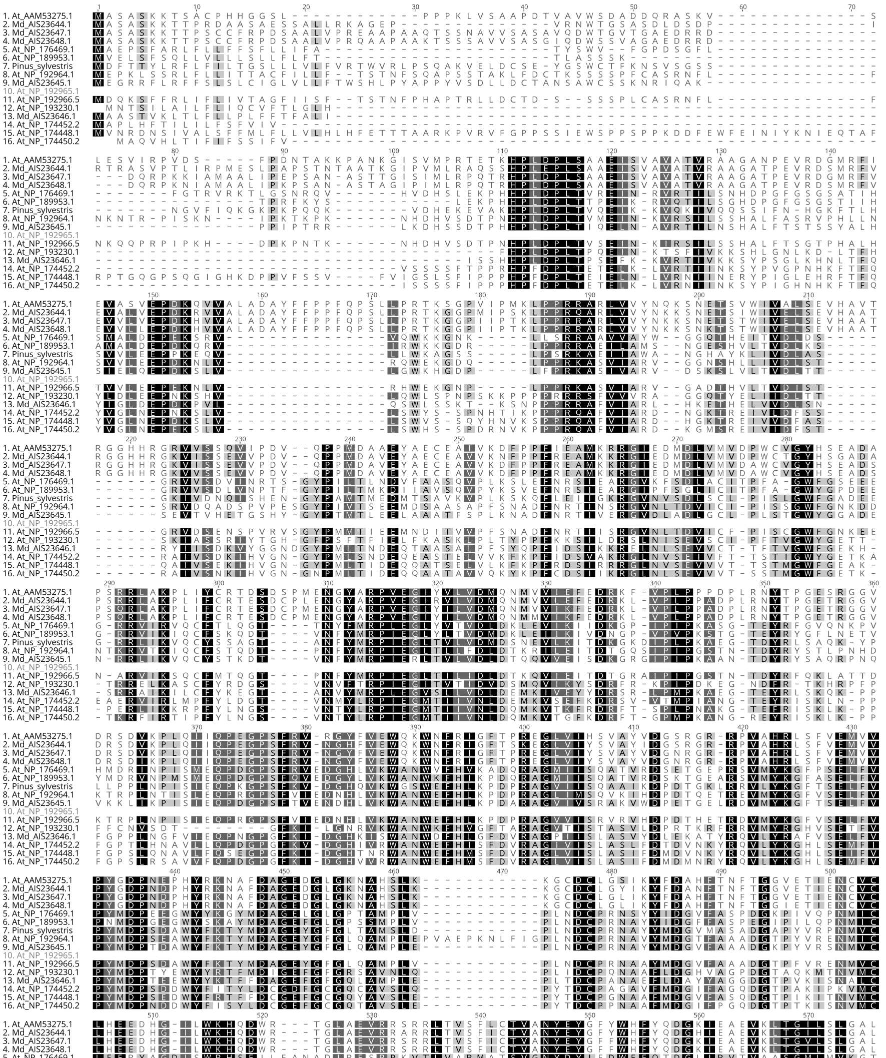

Supplementary Figure S2. Alignment of the CuAO amino acid sequences of *Arabidopsis thaliana*, *Malus domestica* and *Pinus sylvestris*.

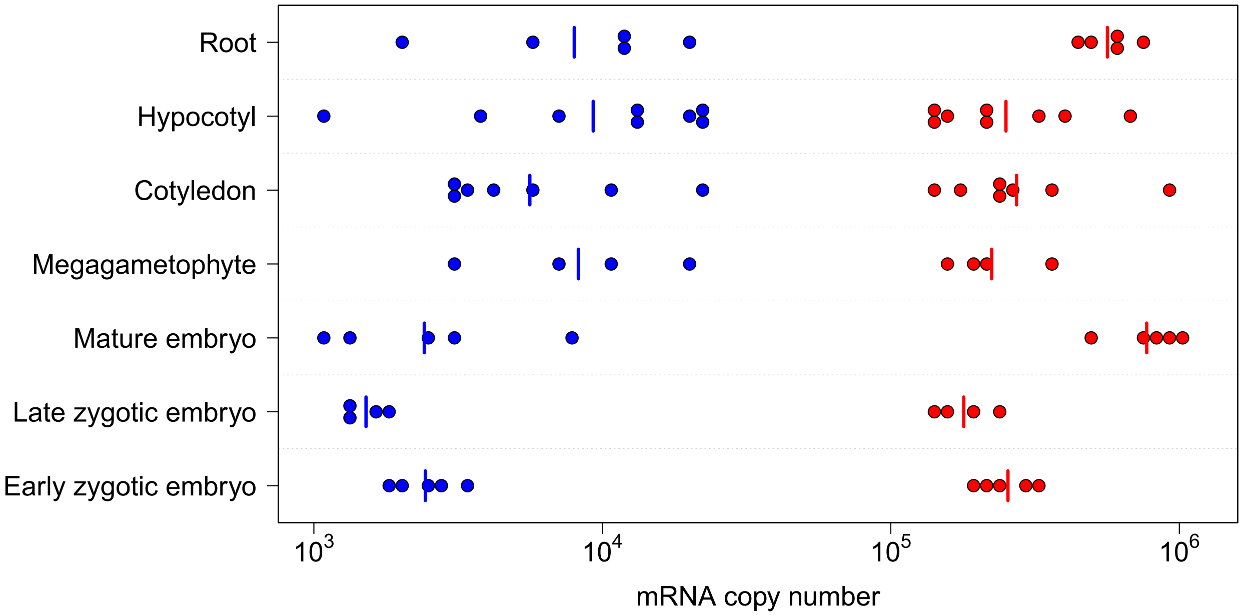

Supplementary Figure S3. The mRNA copy numbers of the arginine decarboxylase *ADC* (red symbols) and ornithine decarboxylase *ODC* (blue symbols) genes in Scots pine tissues. Both *ODC* and *ADC* expression was measured from the same embryo, megagametophyte, cotyledon, hypocotyl and root samples using qPCR with absolute quantification. Each dot represents a single sample i.e. a biological replicate.

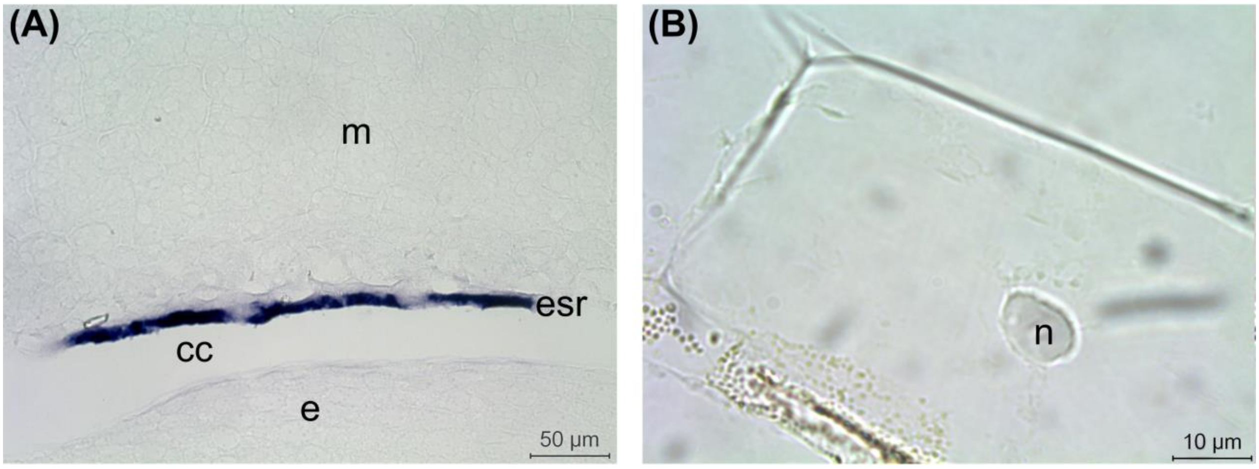

Supplementary Figure S4. Negative controls for *DAO* mRNA *in situ* hybridization assay. (A) A seed section hybridized with the sense *DAO* probe as a negative control showing the non-specific signal in the embryo surrounding region of the megagametophyte. (B) A root section hybridized with the sense *DAO* probe as a negative control.

1.2 Supplementary Tables

Supplementary Table S1. Sequence data for Ka/Ks estimations. The plant species and the NCBI GenBank accession numbers of the sequences used for the calculation of the nonsynonymous to synonymous substitution rate ratios (Ka/Ks) for the thermospermine synthase (*ACL5*), arginine decarboxylase (*ADC*), agmatine iminohydrolase (*AIH*), N-carbamoylputrescine amidohydrolase (*CPA*), diamine oxidase (*DAO*), ornithine decarboxylase (*ODC*), S-adenosyl methionine decarboxylase (*SAMDC*), spermidine synthase (*SPDS*), and spermine synthase (*SPMS*) genes.

Plant Species	*ACL5*	*ADC*	*AIH*	*CPA*	*DAO*	*ODC*	*SAMDC*	*SPDS*	*SPMS*
Apple (*Malus sylvestris*)	AB204521					EU431332			AB204520
Arabidopsis (*Arabidopsis thaliana*)	NM_121958	NM_127204	NM_120900	NM_179770	NM_104959		NM_113454	AJ251296	NM_180848
Black cottonwood (*Populus trichocarpa*)		XM_002306105	XM_002328615	XM_006384731	XM_002329822	XM_002307720	XM_002329275	XM_002315770	
Clementine (*Citrus clementina*)	XM_006452952								XM_024189565
Loblolly pine (*Pinus taeda*)	EST contig	EST contig	EST contig	EST contig	EST contig	EST contig	EST contig	EST contig	
Rice (*Oryza sativa*)	XM_015768360	AY604047	NM_001059567	XM_015769780	NM_001058873	XM_015755390	Y07766	AJ251298	XM_015788948
Scots pine (*Pinus sylvestris*)	HM236828	HM236823	HM236824	HM236825	HM236829	HM236831	HM236826	KX761190	
Sorghum (*Sorghum bicolor*)	XR_002449605	XM_002437713	XM_002446593	XM_002452166	XM_002446165	XM_002462720	XM_002452387	XM_021454016	XM_021449554
Sitka spruce (*Picea sitchensis*)					BT070905	EST contig			
Tobacco (*Nicotiana tabacum*)						Y10472			
Vine (*Vitis vinifera*)		XM_002268994	XM_002269399	XM_002274877	XM_002275836	XM_002281585	AJ567368	XM_002263726	XM_002270754
White spruce (*Picea glauca*)	EST contig	EST contig	EST contig	EST contig			EST contig	BT116397	

Supplementary Table S2. Primers for PCR and sequencing of the *ADC* and *DAO* genes from Scots pine

Forward primer	Primer sequence 5'->3'	Reverse primer	Primer sequence 5'->3'	Type of primer
ADC_Left	GCTCTGAGTCCTGACAGACG	ADC_Right	CCAGCAGTTCGTGATAGATAAACTC	PCR & seq (ADC_Left - ADC_Right)
ADC_F1	ATTTTAACTGTGTTATTGTGCTGGAG	ADC_R1	CAATAACAGGCCTCACAGATAACTT	seq (ADC_Left - ADC_Right)
ADC_F2	GAGGAATTAATGGGTGAATACCAG	ADC_R2	TCTTTGAACAAATCCAGACACCT	seq (ADC_Left - ADC_Right)
ADC_F3	GTGGAGTCTCCCCATCATAACC			seq (ADC_Left - ADC_Right)
ADC_F4	CTGAGTCCTGACAGACGGTAAAAAG			seq (ADC_Left - ADC_Right)
ADC_F5	TTGCTTAGGCGTTGTGGAGT			seq (ADC_Left - ADC_Right)
ADC_F6	AGCGGTTAGTGGTAGATTAGAG	ADC_R6	AGAACCATGAGGTCGAACAG	PCR & seq (F6-R6)
ADC_F7	ACTTGTTCGGTGTGCCCAACGTG	ADC_R7	AGTTCAGAACATGGCTGCTGCAGAT	PCR & seq (F7-R7)
ADC_F8	GGGCTCTCTTCCTGTTCCCCTGG			seq (ADC_Left - ADC_Right)
ADC-Prom_F1	TCTTTTGTCTCACGCATAAATTCACGG	ADC-Prom_R1	CCCCAAATCACAGCACCTCTCAAGC	PCR & seq (ADC-Prom_F1 - ADC-Prom_R1)
ADC-Prom_F2	AAGGGTGTTGCCAGGAAAGTGCG	ADC-Prom_R2	CCAGGGGAACAGGAAGAGAGCCC	PCR & seq (ADC-Prom_F1 - ADC-Prom_R1)
ADC-Prom_F3	GGTTTGTTGTGACTGGAAACCTAAC	ADC-Prom_R3	ACACACCACAAGCCCTCTGCAAC	PCR & seq (ADC-Prom_F1 - ADC-Prom_R1)
DAO_Left	AAGCCATGGATTTCACAACTT	DAO_R2	TTCTAAGAATTGTTTGCTTTTCAGA	PCR & seq (DAO_Left - DAO_R2)
		DAO_R5	GATCTTCTGGACTTTCTTGATCTCC	seq (DAO_Left - DAO_R2)
DAO_F1	GGTGGGCTATATGTTTACCAAAG	DAO_Right	TTCACTCAGATAAAGCTGCACA	seq (DAO_F2 - DAO_Right)

Forward primer	Primer sequence 5'->3'	Reverse primer	Primer sequence 5'->3'	Type of primer
DAO_F2	CAAATTAAGCTGAGCCTCTATCAG	DAO_R14	TCCTTCTCATAGTTGGGTGCAGTCC	seq (DAO_F2 - DAO_Right)
DAO_4F	GACTGACTGCTATGTCTCTTGATCC	DAO_4R	TGAAGCAGATTTAACTTCACTGACA	PCR & seq (DAO_4F - DAO_4R)
		DAO_R5	GATCTTCTGGACTTTCTTGATCTCC	seq (DAO_4F - DAO_4R)
DAO_F6	ATCAAGTCCATAGCTCAAGC	DAO_R6	ACTTCTCGAATCTGTCACAC	seq (DAO_4F - DAO_4R)
DAO_F7	AGCCTGATCCTCGTGCTGGAGTG	DAO_R7	TGAAATTGGACACTCTGTGTGGCG	PCR & seq (DAO_F7 - DAO_R7)
DAO_F8	GGCAGGAAGTAGCCTTCCAAGCAG	DAO_R8	GGGTCCATGTAAGGCACGAAGAGC	PCR & seq (DAO_F8 - DAO_R8)
DAO_F9	GATCCACTCAATGACTGTCCAAG	DAO_R9	GGGTTTGAGTTTTGATTTGACC	seq (DAO_4F - DAO_4R)
DAO_F10	CGAGAAGTCAGACCAAAGGTTAC			seq (DAO_4F - DAO_4R)
DAO-Prom_F1	TGGCAAAATTCCCCAAAATTCTACTCA	DAO-Prom_R1	GCACCAGGACCCATATTCAGAGCA	PCR & seq (DAO-Prom_F1 - DAO-Prom_R1)
DAO-Prom_seqF	GGTAAGCAGAAACCTTGGGTATGGC	DAO-Prom_seqR	CTACCACTGGTGCACCCCAGCAC	seq (DAO-Prom_F1 - DAO-Prom_R1)
DAO_F11	GTCATGGCTAGTGCCGATGGAGC	DAO_R11	TGGTCCTAAGTCACATGGTTTGG	PCR & seq (DAO_F11 - DAO_R11)
DAO_F12	GGGAGGAGCTGCTTTTGCATTCC	DAO_R12	CCTGGGATCTGATCTGGTTAACATTC	PCR & seq (DAO_F12 - DAO_R12)
DAO_F13	TCCTTTCTTGAAGAAGCAACTTGTC	DAO_R13	TGAGGGCATCCAAATCGAAACCTG	PCR & seq (DAO_F13 - DAO_R13)

*PCR indicates the forward and the reverse primers used to obtain the PCR product, and seq indicates the primer used to sequence the obtained PCR product.

Supplementary Table S3. qPCR primers. The sequences of the primers used for the real-time qPCR amplification of the Scots pine polyamine genes: thermospermine synthase (*ACL5*), arginine decarboxylase (*ADC*), agmatine iminohydrolase (*AIH*), N-carbamoylputrescine amidohydrolase (*CPA*), diamine oxidase (*DAO*), ornithine decarboxylase (*ODC*), S-adenosyl methionine decarboxylase (*SAMDC*) and spermidine synthase (*SPDS*) and reference genes: actin (*ACT),* glyceraldehyde-3-phosphate dehydrogenase (*GAPDH*) and ubiquitin (*UBQ*).

Gene	GeneBank Accession No.	Forward primer (5´ → 3´)	Reverse primer (5´ → 3´)	PCR product size
ACL5	HM236828	ACTGCTCACATTCCGTCCTT	TTCGCCTTTGATTCTCTGCT	117
ACT	M36171	GGACAGGTCATTACCGTTGG	GATACCCGCTGCTTCCATT	90
ADC	HM236823	AGTCCGTGTGGCCTGTAATC	TGCACAGACACAACGTCAAA	114
AIH	HM236824	TACCACATGCCTGCTGAATG	TCAGCAAAGACACGTTGACC	110
CPA	HM236825	TTCAGTCCAGGTGACACAGG	CTCTTGCTGCCTCTGGAAAC	100
DAO	HM236829	AATGGGGAAGTTGGGAGTTC	CCCTCCTCAGTTTTCCAGTG	102
GAPDH	L07501	CTGGTGTCTTCACCGACAAA	GGTGCTCATTAACCCCAACA	120
ODC	HM236831	GTGGGTGACTGGCTTGTTTT	GGCATGTAGGCAGCACAGTA	113
SAMDC	HM236826	AAGGAGCAGCTATTTCCACT	CTCCACCAGTGCTTCAAGGT	117
SPDS	KX761190	CCAACGTCCCATTAACCCTA	TGGCAAACAAAATGATGCTG	106
UBQ	AF001948	GAAGGAGCAGTGGAGTCCTG	CAATTTCAGGGACGAGAGGA	104

Supplementary Table S4. The synonymous (Ks) and nonsynonymous (Ka) substitution rates and the Ka/Ks ratios in the thermospermine synthase (*ACL5*) arginine decarboxylase (*ADC*), agmatine iminohydrolase (*AIH*), N-carbamoylputrescine amidohydrolase (*CPA*), diamine oxidase (*DAO*), ornithine decarboxylase (*ODC*), S-adenosyl methionine decarboxylase (*SAMDC*), spermidine synthase (*SPDS*), and spermine synthase (*SPMS*) genes in the selected pairs of angiosperm (A) and gymnosperm (G) plant species.

Pair	Group	Gene	Ks	Ks Std. Err.	Ka	Ks Std. Err.	Ka/Ks
1	G	*ACL5*	0.240	0.042	0.009	0.005	0.038
2	G	*ACL5*	0.004	0.004	0.000	0.000	0.000
3	A	*ACL5*	0.794	0.106	0.110	0.015	0.139
7	A	*ACL5*	1.400	0.348	0.158	0.032	0.113
8	A	*ACL5*	2.158	0.947	0.175	0.088	0.081
9	A	*ACL5*	0.846	0.106	0.136	0.017	0.161
1	G	*ADC*	0.421	0.039	0.035	0.005	0.083
2	G	*ADC*	0.053	0.011	0.009	0.003	0.170
3	A	*ADC*	0.335	0.037	0.044	0.007	0.131
4	A	*ADC*	1.414	0.193	0.160	0.018	0.113
5	A	*ADC*	1.499	0.279	0.139	0.019	0.093
6	A	*ADC*	1.176	0.119	0.104	0.014	0.088
1	G	*AIH*	0.149	0.028	0.032	0.007	0.215
2	G	*AIH*	0.030	0.012	0.006	0.003	0.200
3	A	*AIH*	0.455	0.056	0.067	0.011	0.147
4	A	*AIH*	1.339	0.272	0.159	0.020	0.119
5	A	*AIH*	1.600	0.596	0.154	0.054	0.096
6	A	*AIH*	0.576	0.068	0.111	0.014	0.193
1	G	*CPA*	0.113	0.027	0.030	0.007	0.265
2	G	*CPA*	0.028	0.013	0.006	0.003	0.214
3	A	*CPA*	0.622	0.084	0.034	0.008	0.055
4	A	*CPA*	1.034	0.212	0.085	0.016	0.082
5	A	*CPA*	1.068	0.178	0.086	0.016	0.081
6	A	*CPA*	0.565	0.075	0.047	0.009	0.083
12	G	*DAO*	0.103	0.030	0.036	0.006	0.349
2	G	*DAO*	0.022	0.005	0.001	0.002	0.045
3	A	*DAO*	0.609	1.143	0.089	0.111	0.146
4	A	*DAO*	2.465	0.303	0.178	0.015	0.072

Pair	Group	Gene	Ks	Ks<br>Std. Err.	Ka	Ks<br>Std. Err.	Ka/Ks
5	A	*DAO*	1.833	0.590	0.226	0.035	0.123
6	A	*DAO*	1.033	0.055	0.123	0.012	0.119
12	G	*ODC*	0.296	0.038	0.095	0.012	0.321
2	G	*ODC*	0.069	0.016	0.021	0.005	0.304
3	A	*ODC*	0.418	0.058	0.111	0.014	0.266
6	A	*ODC*	2.831	1.135	0.334	0.165	0.118
10	A	*ODC*	1.983	0.814	0.233	0.090	0.117
11	A	*ODC*	1.311	0.171	0.253	0.023	0.193
1	G	*SAMDC*	0.174	0.028	0.034	0.007	0.195
2	G	*SAMDC*	0.026	0.010	0.007	0.003	0.269
3	A	*SAMDC*	0.795	0.098	0.113	0.015	0.142
4	A	*SAMDC*	1.609	0.575	0.257	0.085	0.160
5	A	*SAMDC*	1.476	0.353	0.227	0.047	0.154
6	A	*SAMDC*	1.060	0.213	0.166	0.027	0.157
1	G	*SPDS*	0.127	0.029	0.009	0.004	0.071
2	G	*SPDS*	0.012	0.009	0.002	0.001	0.167
3	A	*SPDS*	2.829	1.134	0.201	0.102	0.071
4	A	*SPDS*	1.344	0.314	0.074	0.014	0.055
5	A	*SPDS*	1.549	0.619	0.076	0.029	0.049
6	A	*SPDS*	0.455	0.065	0.049	0.010	0.108
3	A	*SPMS*	0.538	0.076	0.067	0.013	0.125
5	A	*SPMS*	1.281	0.286	0.143	0.024	0.112
7	A	*SPMS*	1.422	0.436	0.134	0.033	0.094
8	A	*SPMS*	1.108	0.204	0.115	0.023	0.104
9	A	*SPMS*	0.841	0.120	0.112	0.017	0.133

1 = Scots pine - white spruce
2 = Scots pine - loblolly pine
3 = rice -sorghum
4 = Arabidopsis - black cottonwood
5 = Arabidopsis - vine
6 = black cottonwood -vine
7 = Arabidopsis - clementine
8 = Arabidopsis - apple
9 = clementine - apple
10 = tobacco - black cottonwood
11 = apple - vine
12 = Scots pine - sitka spruce

Supplementary Table S5. Baseline relative gene expressions and estimated relative contrasts (with 95% confidence intervals, CI) during Scots pine zygotic embryogenesis associated with sampling date and Scots pine clones (K818 and K884) and their interactions from fitting a corresponding two-way analysis of variance model with interaction.

	ADC		*AIH*		*CPA*		*DAO*		*SAMDC*		*SPDS*		*ACL5*	
	Est.	95%CI	Est.	95% CI	Est.	95%CI	Est.	95%CI	Est.	95% CI	Est.	95% CI	Est.	95% CI
Baseline: date I, clone K818	0.01	0.00—0.01	0.08	0.05 —0.12	0.00	0.00 —0.01	0.01	0.01—0.02	0.71	0.52—0.99	568.6	385.1 —839.5	0.02	0.01 —0.02
date II	0.71	0.34—1.48	1.54	0.86 —2.76	1.19	0.84 —1.67	0.86	0.48—1.55	0.81	0.51—1.28	0.91	0.53—1.58	0.77	0.47—1.27
date III	1.20	0.57 —2.51	0.96	0.54 —1.72	1.38	0.98—1.93	1.16	0.65—2.07	0.45	0.28—0.71	0.63	0.36—1.09	1.97	1.20—3.24
date IV	1.82	0.87 —3.82	1.00	0.56—1.79	1.09	0.77—1.53	5.25	2.94—9.40	0.64	0.40—1.01	0.60	0.35—1.05	2.38	1.45—3.91
clone K884	0.71	0.34 —1.49	0.96	0.54—1.72	0.62	0.44—0.87	0.51	0.29—0.92	1.19	0.75—1.89	1.39	0.80—2.41	0.55	0.33—0.90
date II:clone K884	1.01	0.35 —2.88	0.76	0.34—1.74	0.81	0.50—1.32	0.95	0.49—2.17	0.75	0.39—1.43	0.44	0.20—0.95	1.20	0.59—2.43
date III:clone K884	0.93	0.27—3.18	1.09	0.42—2.86	0.99	0.56—1.74	4.05	1.54—10.63	0.49	0.21—0.98	0.66	0.27—1.66	1.46	0.65 —3.36
date IV:clone K884	0.46	0.16 —1.37	0.94	0.40—2.20	0.78	0.47—1.28	0.77	0.33—1.79	0.42	0.21—0.82	0.68	0.30—1.52	1.06	0.51—2.18

Log transformation was done before model estimation. The estimated regression coefficients and their 95% CI were back-transformed to the original scale of gene expression.
